# Bilingualism and Performance on Two Widely Used Developmental Neuropsychological Test Batteries

**DOI:** 10.1371/journal.pone.0125867

**Published:** 2015-04-29

**Authors:** Linda C. Karlsson, Anna Soveri, Pekka Räsänen, Antti Kärnä, Sonia Delatte, Emma Lagerström, Lena Mård, Mikaela Steffansson, Minna Lehtonen, Matti Laine

**Affiliations:** 1 Department of Psychology, Åbo Akademi University, Turku, Finland; 2 Niilo Mäki Institute, Jyväskylä, Finland; 3 Department of Psychology, University of Turku, Turku, Finland; 4 Cognitive Brain Research Unit, Cognitive Science, University of Helsinki, Helsinki, Finland; University of Western Ontario, CANADA

## Abstract

The present study investigated the effect of bilingualism on the two widely used developmental neuropsychological test batteries Wechsler Intelligence Scale for Children – Fourth Edition (WISC-IV) and A Developmental Neuropsychological Assessment, Second Edition (NEPSY-II) in children. The sample consisted of 100 Finland-Swedish children in two age groups. About half (n = 52) of the participants were early simultaneous bilinguals, and the other half (n = 48) were monolinguals. As no Finland-Swedish versions of the tests are available at the moment, both tests were translated and adapted to suit this population. The results revealed no difference in the performance between bilingual and monolingual children. This speaks against a cognitive advantage in bilingual children and indicates that development of separate norms for monolingual and bilingual children is not needed for clinical use.

## Introduction

When investigating cognitive development in children, researchers have found an advantage in bilinguals concerning certain cognitive abilities, especially nonverbal executive control and theory of mind [1–3]. The reason for a possible advantage is thought to stem from the fact that managing two languages requires executive resources for selecting the relevant language and inhibiting the language not in use at the moment [4–5]. Some studies argue that the advantage for bilinguals arises when the task has higher executive demands [2, 6–7], and when the bilinguals are balanced in their two languages [8–9]. There is, however, a growing body of studies indicating that the results regarding a bilingual advantage are inconsistent and that there has been a clear publication bias towards studies showing significant results [10–11]. Some also argue that the bilingual advantage can be explained by other factors, such as socioeconomic status and small sample sizes [12–13]. Recent large-scale studies with well-matched groups have not found support for the hypothesis of a bilingual advantage [14–17]. Paap, Johnson, and Sawi [13] compared individuals with different levels of bilingualism, but the bilingual groups were not better than the monolingual group on any of the executive function tasks. In verbal tasks, however, monolinguals have been shown to perform better than bilinguals [4, 18–20]. This may partially be due to the fact that monolinguals possess a larger vocabulary in the language that is investigated than bilinguals do [21]. Statistically controlling for this group difference in verbal performance may bring up differences in executive performance that are more apparent than real (see [22]).

Despite the earlier literature suggesting an executive advantage in bilinguals, few studies have investigated the clinical relevance of bilingualism to neuropsychological testing. Among the most frequently used neuropsychological tests for children are the Wechsler Intelligence Scale for Children—Fourth Edition (WISC-IV [23]) and A Developmental Neuropsychological Assessment, Second Edition (NEPSY-II [24]). Studies using WISC or NEPSY to assess differences in performance between bilingual and monolingual children have only administered some of the subtests, and the results have not been fully in line with the general findings regarding bilingualism and cognitive ability. Bialystok and Majumder [8] used the subtest Block Design from WISC-R [25] to measure 7- to 9-year-old monolingual and bilingual children’s ability to perceive and analyze patterns, and found that bilinguals received higher scores than monolinguals in the task. Lauchlan, Parisi, and Fadda [26] administered four WISC subtests [27–28], Block Design, Digit Span, Vocabulary, and Arithmetic to mono- and bilingual children (mean age range for the different groups: 9 years and 1 month-10 years and 4 months), and found an advantage for bilingual children in Block Design and, quite surprisingly, in Vocabulary. No significant group differences emerged for the Digit Span or Arithmetic subtests. Korkman et al. [29] used the NEPSY [30] to investigate 5- to 7-year-old bilingual and monolingual Finland-Swedish children’s verbal capacity. Of the subtests administered (Body Part Naming, Speeded Naming, Comprehension of Instructions, Repetition of Nonsense Words, Narrative Memory, and Sentence Repetition), the bilingual children scored significantly lower on Body Part Naming. Garratt and Kelly [31] compared the performance of 6- to 7-year-old monolingual and bilingual children, unbalanced in their two languages, on the 14 core subtests of NEPSY [32]. The bilinguals scored significantly lower than the monolinguals in the two verbal subtests Speeded Naming and Comprehension of Instructions. The bilinguals also scored significantly lower than monolinguals in Visual Attention. In contrast, the bilinguals outperformed the monolinguals on Imitating Hand Positions and Design Copying. No significant differences in performance between the groups were found on the remaining NEPSY core subtests (Tower, Auditory Attention, Phonological Processing, Finger Tapping, Visuomotor Precision, Arrows, Memory for Faces, Memory for Names, Narrative Memory).

Given the variable results from the small number of available studies reviewed above, it is evident that more research is needed to study the possible effects of bilingualism on widely used developmental cognitive test measures. Firstly, this is relevant for the clinical use of these test instruments where the test results of a child can have important diagnostic consequences. In borderline cases, a possible positive or negative effect of bilingual language background that the diagnostician is unaware of could tip the balance of a clinical decision in one direction or the other. Secondly, albeit standardized cognitive test batteries are not designed to tap functions specifically associated with bilingualism, they can provide evidence relevant to the issue of a possible bilingual executive advantage. Accordingly, we investigated how bilingualism affects the performance of two age groups of Finland-Swedish children on selected subtests of the two widely used neuropsychological tests Wechsler Intelligence Scale for Children—Fourth Edition (WISC-IV) and A Developmental Neuropsychological Assessment, Second Edition (NEPSY-II). Finland-Swedish bilingual children provide a particularly interesting study group, as they grow up in an officially bilingual society and their culture is very close to that of their monolingual Finnish-speaking peers. Children have equal opportunities to use their preferred home language as a day care and school language. Likewise, books, newspapers and TV programs are accessible in both languages. Thus, potentially confounding effects, such as cultural and socioeconomical factors, often present between a language majority and minority, are minimized in the present group comparison.

## Materials and Methods

### Participants

The sample consisted of 100 Swedish-speaking children recruited through public schools in Finland. The children represented two separate age groups; 50 children were 7 years and 1 month to 7 years and 6 months, while 50 children were 10 years and 10 months to 11 years and 2 months. In the younger sample, 25 (50%) children were bilingual and 25 (50%) monolingual, while 27 (54%) were bilingual and 23 (46%) monolingual in the older sample. All of the monolinguals were Swedish speakers. Of the bilingual children, 48 were reported by their parents to have acquired both languages by the age of three, and all bilingual children had learned their second language by the age of six. One child in the younger age group, who was reported to speak three languages fluently, was excluded from the analyses. Information regarding the language abilities of the bilinguals is presented in Table 1.

**Table 1:** Language abilities of the bilingual children.

		Younger	Older	Total
		*n*	*n*	*n*
Bilinguals		24	27	51
Swedish and Finnish		21	22	43
	Swedish stronger	10	12^a^	22^a^
	Finnish stronger	5	3^a^	8^a^
	Both equally strong	6	6^a^	12^a^
Swedish and other language		3	5	8
	Swedish stronger	2	3	5
	Other language stronger	1	0	1
	Both equally strong	0	2	2

^a^Information on one participant missing.

In order to ensure geographical representativeness, stratified sampling was used. The Swedish-speaking area of Finland was divided into three main geographical regions; the Helsinki Capital region, Ostrobothnia, and the remaining Swedish-speaking areas in southern Finland (encompassing coastal areas east and west of the Capital region). The Åland Islands were not included due to the similarity of its language culture with that of Sweden. Information from the reporting portal of the Finnish National Board of Education (Vipunen [33]) was used to determine the number of children needed from each region.

Subsequently, a random sampling of six schools from each region was carried out. Schools with less than 12 students at the 1^st^, 2^nd^ or 3^rd^ grade in 2011 according to the Finnish National Board of Education [33] were excluded due to practical reasons. Private schools, language immersion schools, and schools for children with special needs were also excluded. The directors of education in the municipalities of the resulting 18 schools were contacted and after their approval, the headmasters of each school were contacted, and letters were sent out to the parents of all 1^st^, 4^th^ and 5^th^ graders. The letter contained information about the study and two forms for the parents of the children to fill out, namely a background information form and an informed consent. Due to non-responders and the need to exclude one school in Ostrobothnia that turned out to have a class size smaller than 12, reminder letters were sent out. Also, four of the schools in the Capital region declined participation. Thus, another school in the Capital region was randomly selected and contacted. The total amount of letters sent out was 990, of which 359 (36.3%) were returned. Of these returned letters, 314 (87.5%) accepted participation in the study. Children outside the specific age range of the study were excluded, as well as children with diagnosed psychiatric or neurological disorders. Remedial instruction/education did not serve as exclusion criterion. As more letters than needed were returned, the inclusion order was based on the randomization order of the schools and the date the letters were returned. See Table 2 for a description of the sample.

**Table 2:** Background data on the participants.

	Younger		Older	
	Monolingual	Bilingual	Monolingual	Bilingual
	*n* = 25	*n* = 24	*n* = 23	*n* = 27
**Age in years**				
mean	7.3	7.4	11.0	11.0
range	7.1–7.5	7.1–7.5	10.8–11.2	10.8–11.2
**Gender**				
male	13	16	8	17
female	12	8	15	10
**Handedness**				
right	21	21	19	21
left	4	2	3	5
both	0	1	1	1
**Remedial instruction** ^a^				
Yes	3	4	4	5
No	20	20	19	21
**Mother educational level** ^a^				
level 1	1	0	0	0
level 2	8	6	10	10
level 3	6	5	6	4
level 4	10	12	7	11
**Father educational level** ^a^				
level 1	2	0	1	2
level 2	12	7	6	8
level 3	8	3	7	3
level 4	3	13	9	12

*Note*. One of the bilinguals in the younger sample, who was reported to speak three languages fluently, was excluded; Mother educational level = highest educational level attained by the mother of the child; Father educational level = highest educational level attained by the father of the child; Level 1 = Comprehensive school; Level 2 = Upper secondary education or vocational education; Level 3 = Lower-degree level tertiary education; Level 4 = Higher-degree level tertiary education or doctorate.

^a^Information on this point was not obtained from some participants.

### Measures and procedure

The measures used in the present study were the Wechsler Intelligence Scale for Children—Fourth Edition (WISC-IV) and A Developmental Neuropsychological Assessment, Second Edition (NEPSY-II). For WISC-IV, all subtests except the supplementary ones were administrated. For NEPSY-II, a subtest was included in the battery if it contained a language component or had a Cronbach’s alpha reliability value of more than .7 for the selected age groups. The reliability values used were those reported in the Finnish version of NEPSY-II [16]. The selected subtests belonged to the domains Alertness and Planning, Linguistic Function, and Memory and Learning. All administered subtests and the cognitive components they are thought to measure are presented in Table 3. The data collection was carried out from January to April 2014 by five advanced psychology students trained in administering the measures. The children were tested individually in a single session, in a room provided by their own school. The session lasted approximately 3 hours (1.5 hours per test), and the test order (WISC-IV, NEPSY-II) was counterbalanced.

**Table 3:** The subtests used in the present analyses and the cognitive components they are thought to measure [23–24].

Subtest	Cognitive component
**WISC-IV**	
Similarities	Verbal reasoning and concept formation
Vocabulary	Word knowledge, verbal concept formation, level of language development
Comprehension	Verbal comprehension and reasoning, capacity to evaluate and utilize one’s own past experiences
Block Design	Visuospatial processing, nonverbal concept formation
Picture Concepts	Abstract categorical reasoning
Matrix Reasoning	Visual information processing and nonverbal reasoning
Digit Span	Auditory short-term and working memory
Letter-Number Sequencing	Auditory working memory
Coding	Speeded visual processing, short-term memory, visuo-motor coordination
Symbol Search	Speeded visual discrimination
**NEPSY-II**	
Auditory Attention A	Selective and sustained attention
Auditory Attention B	Set shifting and maintaining, inhibition of previously learned responses, and working memory
Comprehension of Instructions	Ability to receive, process, and execute oral instructions
Phonological Processing	Phonemic awareness
Speeded Naming	Lexical access and automaticity of verbal information
Word Generation	Language acquisition and retrieval, working memory, speed of processing, attention, and verbal productivity
Memory for Names	Acquisition and retrieval of verbal labels in a task of visual-verbal paired-associate learning
Narrative memory	Memory for organized verbal material
Word List Interference	Verbal working memory, repetition, and word recall following interference

#### Translation of the measures

As neither of the tests used in the present study have been adapted for the Swedish-speaking population of Finland, the Finnish versions of both WISC-IV [23] and NEPSY-II [24] were translated into Finland-Swedish versions. As the language in Sweden differs from the Finland-Swedish one, the Swedish versions of the tests [34–35] could not be used. However, when appropriate, the Swedish versions of the tests were used as support in the translation process. Psychologists, teachers, researchers, and linguists were involved in the translation process, and the resulting test was piloted on a small number of children.

For all subtests, the test instructions and verbal scoring criteria were translated from Finnish into Swedish. With regard to WISC-IV Similarities, the direct translations for two of the items turned out to have multiple meanings in Swedish. Therefore, these items were changed to other words that were considered equivalent. In the same vein, seven items in the Vocabulary subtest were altered due to linguistic and cultural reasons. Concerning NEPSY-II, the Swedish version of the accompanying CD in NEPSY [30] was used for the subtests Auditory attention and response set A and B. Also for Phonological processing, the Swedish version of the subtest was used, and in the phonological part of Word generation, the letters from the Swedish version were presented to the children. In Memory for names, the cards were taken from the Finnish version of NEPSY- II, while the names were taken from the Swedish version. For Narrative memory, the story was translated into Swedish from the Finnish version of NEPSY-II, but some words, such as names, were taken from the Swedish version of NEPSY-II.

### Ethics Statement

The study was approved by the Institutional Review Board of the Department of Psychology and Logopedics at Åbo Akademi University. The parents of the children signed a written informed consent prior to participation.

### Missing values

Due to administration errors (some of the subtests were terminated before the criterion of number of errors were reached), scores for 33% of the children in Vocabulary, 18% in Letter-Number Sequencing, 9% in Comprehension, and 25% in Word List Interference, were not available for the statistical analysis, but replaced with imputed values. The scores were assumed to be missing at random, and multiple imputation was therefore used to handle the missing data. In the imputation process, several versions of the dataset were generated with the fully conditional specification, an iterative Markov chain Monte Carlo (MCMC) method in the IBM SPSS Statistics (version 21) Multiple Imputation module. Graphical diagnostics suggested that 400 iterations were sufficient to reach convergence, so a dataset was saved every 400^th^ iteration until 100 filled-in copies of the dataset had been computed. To avoid generating outlying values, imputed values were constrained to stay within the ranges specified by the manuals for the tests. The raw scores from all the administered subtests in both WISC-IV and NEPSY-II were used as predictors. The subsequent analyses on imputed variables were performed separately on each complete dataset, and Rubin's [36] rules were used to summarize the parameter estimates and their standard errors into a single set of results. These pooled statistics will be reported when any of the variables containing imputed values are included in the analyses.

## Results

In order to ensure that the two language groups were balanced on gender, remedial teaching and parent educational level, two-sided Fisher’s exact tests were conducted separately for each age group. In these analyses, no differences with regard to gender, remedial teaching, or the educational levels of the mothers were found between the monolingual and bilingual children in neither age group. However, a difference in the educational levels of the fathers between the language groups was found in the younger age group (*p* = .003). The fathers of the bilingual children had a higher level of education than the fathers of the monolingual children. Only the statistics of significant results involving the language groups will be reported here.

The average raw scores of the monolinguals and bilinguals on each administered subtest are presented separately for each age group in Tables 4 and 5. To investigate the effect of bilingualism on test performance, ANOVAs were conducted. In these analyses, the raw score of each subtest, or the standardized scores of each index, served as dependent variables. The independent variables were language group (monolingual; bilingual) and age group (younger; older). For imputed datasets, IBM SPSS Statistics (version 21) does not provide pooled *F*-statistics for ANOVAs. Therefore, linear regression analyses were conducted when the analyses included variables containing imputed values. The dependent variables in these analyses were the raw score of the subtests or the standardized scores of the indexes, while language group (monolingual; bilingual) and age group (younger; older) were used as predictors.

**Table 4:** Test Performance in Raw Scores of Monolinguals and Bilinguals in the younger sample.

	Monolinguals (n = 25)			Bilinguals (n = 24)		
	*range*	*M*	*SD*	*range*	*M*	*SD*
**WISC-IV**						
Similarities	6–20	13.1	4.2	0–21	12.2	4.3
Vocabulary		15.9	5.9		18.4	5.2
Comprehension	4–32	18.6	6.7	6–28	19.1	5.4
Block Design	14–46	24.6	7.9	10–40	25.2	7.9
Picture Concepts	7–20	14.8	3.1	7–19	14.6	3.2
Matrix Reasoning	7–24	15.1	5.3	3–22	13.9	4.9
Digit Span	8–16	12.1	2.0	8–15	11.2	1.8
Letter-Number Sequencing		10.9	4.8		10.6	4.5
Coding	25–60^a^	42.2^a^	9.9^a^	23–61	42.0	11.0
Symbol Search	19–33	26.4	3.7	14–35	21.9	5.3
**NEPSY-II**						
Auditory Attention A	15–60^a^	52.7^a^	9.5^a^	25–60^b^	51.2^b^	8.5^b^
Auditory Attention B	16–66	49.3	11.8	31–68	52.7	9.4
Comprehension of Instructions	17–29	23.2	3.1	16–31	21.6	3.9
Phonological Processing	22–41	30.4	5.7	16–42	29.5	7.4
Speeded Naming, time	92–286^a^	194.4^a^	54.5^a^	130–360	198.7	50.2
Word Generation, Total	16–44	28.9	8.0	17–54	30.8	11.4
Word Generation, Semantic	10–33	19.8	6.1	11–38	22.3	7.4
Word Generation, Phonemic	3–19	9.0	4.1	2–21	9.3	6.1
Memory for Names	1–27	13.2	5.8	2–27	11.2	6.1
Narrative Memory	7–37	24.1	8.9	12–36	23.8	6.9
Word List Interference		28.8	7.8		28.2	7.3

*Note*. Ranges for the subtests including imputed values are not presented.

^a^Information on one participant missing.

^b^Information on two participants missing.

**Table 5:** Test Performance in Raw Scores of Monolinguals and Bilinguals in the older sample.

	Monolinguals (n = 23)			Bilinguals (n = 27)		
	*range*	*M*	*SD*	*range*	*M*	*SD*
**WISC-IV**						
Similarities	15–28	22.1	3.6	15–37	22.8	4.7
Vocabulary		31.8	8.4		32.6	6.7
Comprehension		32.1	4.5		31.8	5.2
Block Design	26–56	41.8	9.4	21–58	40.8	11.1
Picture Concepts	15–24	20.0	2.4	16–22	19.2	1.9
Matrix Reasoning	15–28	23.3	3.1	13–30	22.0	4.2
Digit Span	11–20	14.6	2.2	12–20	14.7	1.9
Letter-Number Sequencing		17.5	1.8		18.3	1.8
Coding	28–62	45.3	8.3	24–76	43.6	10.2
Symbol Search	20–30	25.3	2.7	16–32	24.8	4.3
**NEPSY-II**						
Auditory Attention A	54–60	58.5	2.0	54–60	58.1	2.1
Auditory Attention B	55–72	66.0	4.4	44–71	64.7	6.1
Comprehension of Instructions	21–33	28.4	2.9	19–33	27.6	4.0
Phonological Processing	33–45	40.4	3.4	32–45	40.4	3.1
Speeded Naming	30–55	41.8	6.8	28–57	43.6	7.3
Word Generation, Total	33–76	52.9	10.6	29–87	50.0	14.3
Word Generation, Semantic	21–55	34.4	8.7	17–49	31.0	7.6
Word Generation, Phonemic	4–27	18.5	5.7	5–38	18.7	8.1
Memory for Names	12–30^a^	21.0^a^	5.2^a^	11–29	21.3	4.8
Narrative Memory; story 2	25–40	34.1	4.5	14–38	30.6	7.0
Narrative Memory; story 3	2–17	9.7	4.7	2–22	11.1	5.8
Word List Interference		35.5	5.6		33.9	6.1

*Note*. Monolinguals, story 2 n = 9, story 3 n = 14; Bilinguals, story 2 n = 16, story 3 n = 11; Ranges for the subtests including imputed values are not presented.

^a^Information on one participant missing.

The ANOVA on Symbol Search revealed a significant main effect of language group, *F*(1, 95) = 9.30, *p* = .003, η^2^_partial_ = .089, and a significant interaction between language and age, *F*(1, 95) = 5.85, *p* = .017, η^2^_partial_ = .058. The results indicated that in the younger age group, the monolinguals received higher scores than the bilinguals. No other statistically significant effects of language group were found in any of the analyses.

## Discussion

The present study investigated whether bilingualism affects school-aged children’s performance on selected subtests of the two widely used neuropsychological tests Wechsler Intelligence Scale for Children—Fourth Edition (WISC-IV) and A Developmental Neuropsychological Assessment, Second Edition (NEPSY-II). The sample consisted of 100 Finland-Swedish children in two age groups, 7-year-olds and 10- to 11-year-olds.

Overall, our results indicated that the bilingual vs. monolingual language background do not affect children’s performance on these cognitive test batteries. The only statistical difference was that the monolinguals scored significantly higher than the bilinguals on the subtest Symbol Search in WISC-IV. This difference was found only in the younger age group.

Previous research on WISC has found bilinguals to perform better than monolinguals on Block Design and Vocabulary [8, 26]. However, Bialystok and Majumder [8] compared an English-speaking monolingual group to a French–English bilingual and a Bengali-English bilingual group. The school language was French in one of the groups and English in the two others and the tests were administered in English. Also, Bialystok and Majumder [8] did not take socioeconomic status into account. Lauchlan, et al. [26] compared bilingual children from Scotland and Sardinia to monolingual English and Italian speaking children. The difference in vocabulary favoring the bilingual children was found only for the Scottish group but not for the Sardinian children. Therefore, the group differences reported by Lauchlan et al. [26] and Bialystok and Majumder [8] may stem from other factors than bilingualism.

Regarding previous studies comparing bilinguals and monolinguals on NEPSY, the present results are in line with the Finnish study by Korkman et al. [29], where no difference in performance was found between monolinguals and bilinguals on the subtests Speeded Naming, Comprehension of Instructions, and Narrative Memory of the NEPSY-II test battery. Of the subtests administered in the study by Garratt and Kelly [31], the present study administered Auditory Attention, Phonological Processing, Speeded Naming, Comprehension of Instructions, Memory for Names, and Narrative Memory. In contrast to the results from the present study and the study by Korkman et al. [29], the performance of the bilingual children in the Garratt and Kelly [31] study was weaker than that of the monolinguals on the two verbal subtests Speeded Naming and Comprehension of Instructions.

The present study did not find significant performance differences in favor of the bilingual group and therefore does not support previous studies showing a bilingual advantage in children. It should also be noted that the results from previous research have been inconsistent regarding a bilingual advantage [1–2, 11, 13]. De Bruin, Treccani, and Della Sala [10] suggest that there has been a clear publication bias towards studies showing significant results. Some also argue that the bilingual advantage can be explained by other factors, such as socioeconomic status and small sample sizes [12–13]. The question of whether there is a bilingual advantage has thus not yet been answered, despite the great number of studies investigating the issue (see, e.g., [1–2; 11, 13]).

The bilingual advantage is typically seen in tasks measuring executive functions, such as inhibition of irrelevant information and working memory [1–2]. The test batteries employed here mainly include subtests that are not primarily executive (although performance in all subtests naturally enough requires some executive processes). The ones with the greatest demands on executive functioning are Auditory Attention A (attention) and B (set shifting, inhibition, and working memory), Word Generation (attention, working memory), Word List Interference (working memory), Digit Span (working memory) and Letter-Number Sequencing (working memory), but no group differences were seen on these subtests either.

In verbal tasks, monolinguals have often been shown to perform better than bilinguals [4, 18–20]. In the present study, the bilinguals had learned both languages before the age of 7, and the tests were administered in the same language as they used in school, which may explain why no differences in any verbal subtests between the language groups were found.

The single significant result indicating an advantage for monolinguals on the subtest Symbol Search that taps perceptual speed has not been reported previously and escapes a clear theoretical explanation in the present context. It may, thus, represent a chance finding as it was the only significant group difference, observable in one age group but not in the other, and difficult to explain in the light of previous research on bilingualism and cognitive function.

The effects of bilingualism on cognitive performance remain a hot research topic. In sum, the results from the present study show no difference between monolingual and bilingual children on selected subtests of WISC-IV and NEPSY-II. The main implication of our study for clinical psychological practice is that for diagnostic use, there is no need for separate norms for monolingual and bilingual children.
